# Data-driven optimisation of variation of residual concentration to estimate the hydrogen diffusion coefficient and uptake via MATLAB

**DOI:** 10.1016/j.dib.2025.111891

**Published:** 2025-07-21

**Authors:** Luca Gritti, Denny Coffetti, Marina Cabrini, Tommaso Pastore

**Affiliations:** University of Bergamo, Bergamo, Italy

**Keywords:** Data-driven optimisation, Hydrogen modelling, Hydrogen uptake, Apparent diffusion coefficient, Discharging target curve

## Abstract

This article presents the data collected during experimental solubility tests in University of Bergamo lab and the developed code to estimate the principal parameters for hydrogen uptake. The experimental approach proposed involves in two-step process: the first phase consists of electrochemical charging to saturate the metallic sample via cathodic polarization at potentials lower than the equilibrium potential for hydrogen evolution, followed by a second phase of discharging under anodic polarization at potentials higher than the equilibrium potential. During the discharging phase, the time-dependent anodic current is influenced by the flux of diffusible hydrogen exiting the sample. This flux is governed by the initial concentration of diffusible hydrogen in the material, the diffusion coefficient, and the time elapsed between the end of the charging step and the beginning of the discharging phase. Via a data-driven optimisation it is possible to obtain the characteristic parameter of hydrogen diffusion in the material (apparent diffusion coefficient, hydrogen uptake and waiting time) using the MATLAB code. The data permit to elaborate the cylindrical geometry, however it is possible to modify the target curve via a simulation on a specific geometry (not included in the data) and use the same method to elaborate the experimental data of a specific geometry.

Specifications Table

**Table:** 

Subject	Engineering & Materials science
Specific subject area	Modelling diffusion hydrogen on tri-dimensional fluxes, investigation of principal parameters about hydrogen in metals after electrochemical charging. Estimation of hydrogen diffusion coefficient, uptake and waiting time (between end of electrochemical charging and start of discharging).
Type of data	.mat (MATLAB code) .txt (target curve for specific cylindrical geometry) .xlsx (experimental data and pre-processed data)
Data collection	The data of target curve are collected via COMSOL simulations reordering all simulation curves obtained by [1] . The experimental curves are obtained detecting the electrochemical discharging circulating current. Via the MATLAB code it is possible elaborated the experimental data as described in [1]
Data source location	Data collected and elaborated in Laboratories of University of Bergamo via Galvani, 2, 24,044 Dalmine (Bg), Lombardy, Italy
Data accessibility	Repository name: **Data in Brief: Experimental Data Supporting Solubility Model** Data identification number: 10.17632/c239nmw6dp.1 Direct URL to data: data.mendelay.com Gritti, Luca; Coffetti, Denny; Cabrini, Marina; Pastore, Tommaso (2025), “Data in Brief: Experimental Data Supporting Solubility Model”, Mendeley Data, V1, doi: 10.17632/c239nmw6dp.1
Related research article	G. Luca, C. Denny, Marina Cabrini, Tommaso Pastore, A novel approach for estimating hydrogen uptake and diffusion coefficient, Int J Hydrogen Energy (n.d.).

## 1 Value of the Data

• The experimental data can be used to verify the code, represents the experimental results of electrochemical discharging curve of hydrogen and average residual concentration.
• The target curve data can be used to estimate the diffusion coefficients and solubility of hydrogen for the specific cylindrical specimens in electrochemical solubility tests.
• The MATLAB code elaborates the preprocess concentration discharging curve via data driven optimization for estimate diffusion coefficient, hydrogen uptake and waiting time between electrochemical charge/discharge.
• By estimating another target curve for a specific geometry, it is possible to use this MATLAB code to extrapolate the diffusion coefficient, solubility and waiting time between electrochemical charge/discharge.

## 2 Background

Thank this data it is possible elaborate directly the experimental data detected via method explained in the paper “A novel approach for estimating hydrogen uptake and diffusion coefficient” [1]. The data driven optimisation code can be employed to estimate the initial hydrogen concentration C_0_, the diffusion coefficient D, and the time interval t_0_ between the end of the charging phase and the start of the initial discharging phase based on experimental electrochemical discharging curves as explained in [1]. The experimental data should be normalized as described in [1] by assigning the appropriate values to C_0_, D, and t_0_. The normalized experimental data can be adjusted to align with the reference target curve by modifying the normalization parameters C_0_, D, and t_0_. Optimization is employed to minimize the relative error function, which is computed as the difference between the two curves. The procedure was implemented using MATLAB code. The experimental curves and MATLAB procedure is accessible on Mendeley Data [2]. The iterative process utilizes the *fminsearch* function, a nonlinear optimization solver based on the Nelder–Mead simplex algorithm. This method involves a simplex of *n* + 1 points, ordered by the decreasing value of the objective function. The worst point is discarded and replaced by a new point according to specific criteria.

## 3 Data Description

**Fig. 1:**
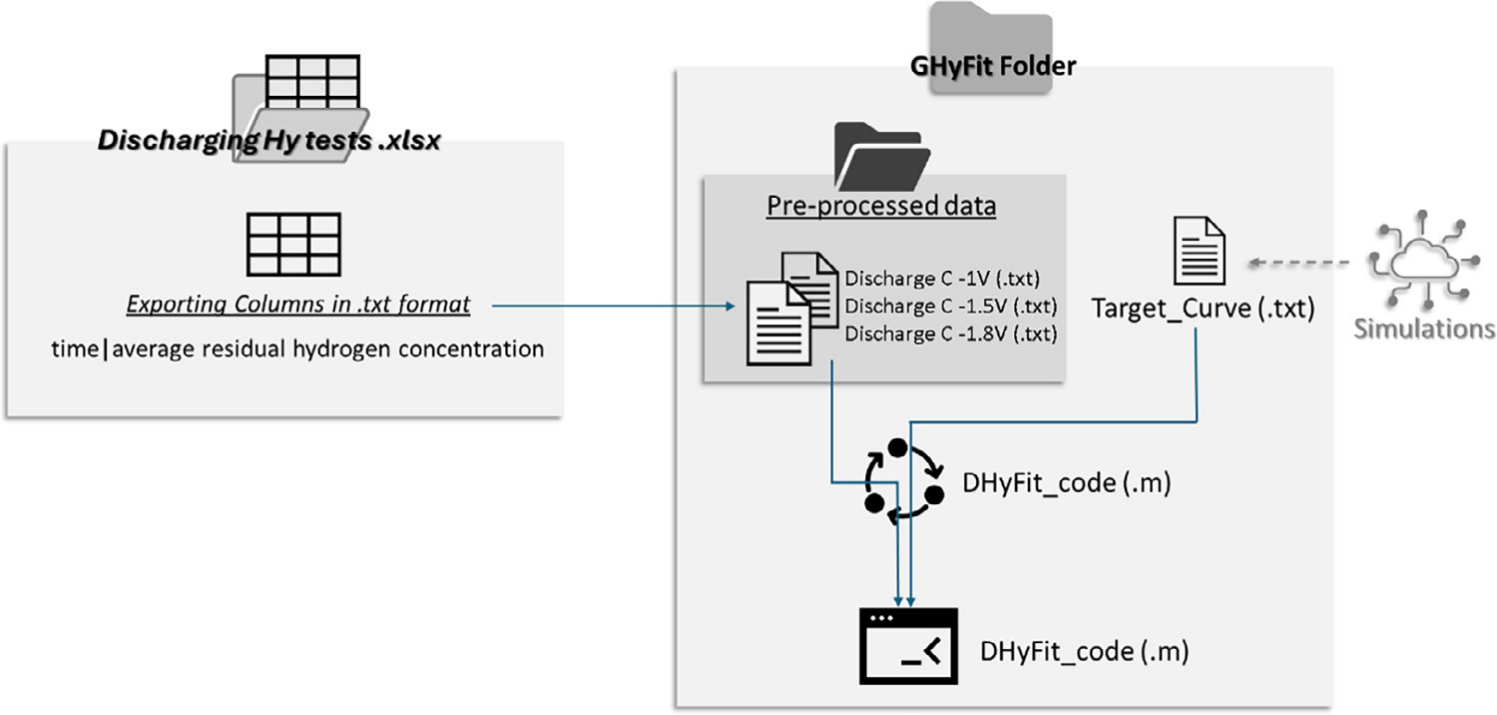
Scheme of procedure and location of data.

The data are available in the Mendeley Data “**Data in Brief of Solubility Model”.** The data is arranged in the following folders, in Fig. 1 it is shown a flow chart of procedure:

1) **Discharging Hy tests (.xlsx)**
  This is the excel file with experimental data collected by solubility tests. In the file there are experimental data (time and current) for different charging cathodic polarizations (−1, −1.5, −1.8 V vs SCE). Are reported the pre-elaboration curves: from curve of current measured it is estimated the residual average concentrations as describe in [1].
2) **HyFit Folder**
  ○ HyFit_code (.m)
    This is the MATLAB code to elaborate the data. In this part there is data driven optimization. The code is commented line by line.
  ○ Importfile (.m)
    It is a function to import the data in format .txt in the code
  ○ Preprocessed data (.txt)
    This is the data to import in the MATLAB code for elaboration. Each file is composed of two columns, acquisition time and residual average concentration (estimated by excel file)
  ○ Target_Curve (.txt)
    This file contains the target curve elaborated by COMSOL simulations described in the article [1]

## 4 Experimental Design, Materials and Methods

■ **EXPERIMENTAL DATA, CURVES AND PRE-PROCESSED DATA**

**Fig. 2:**
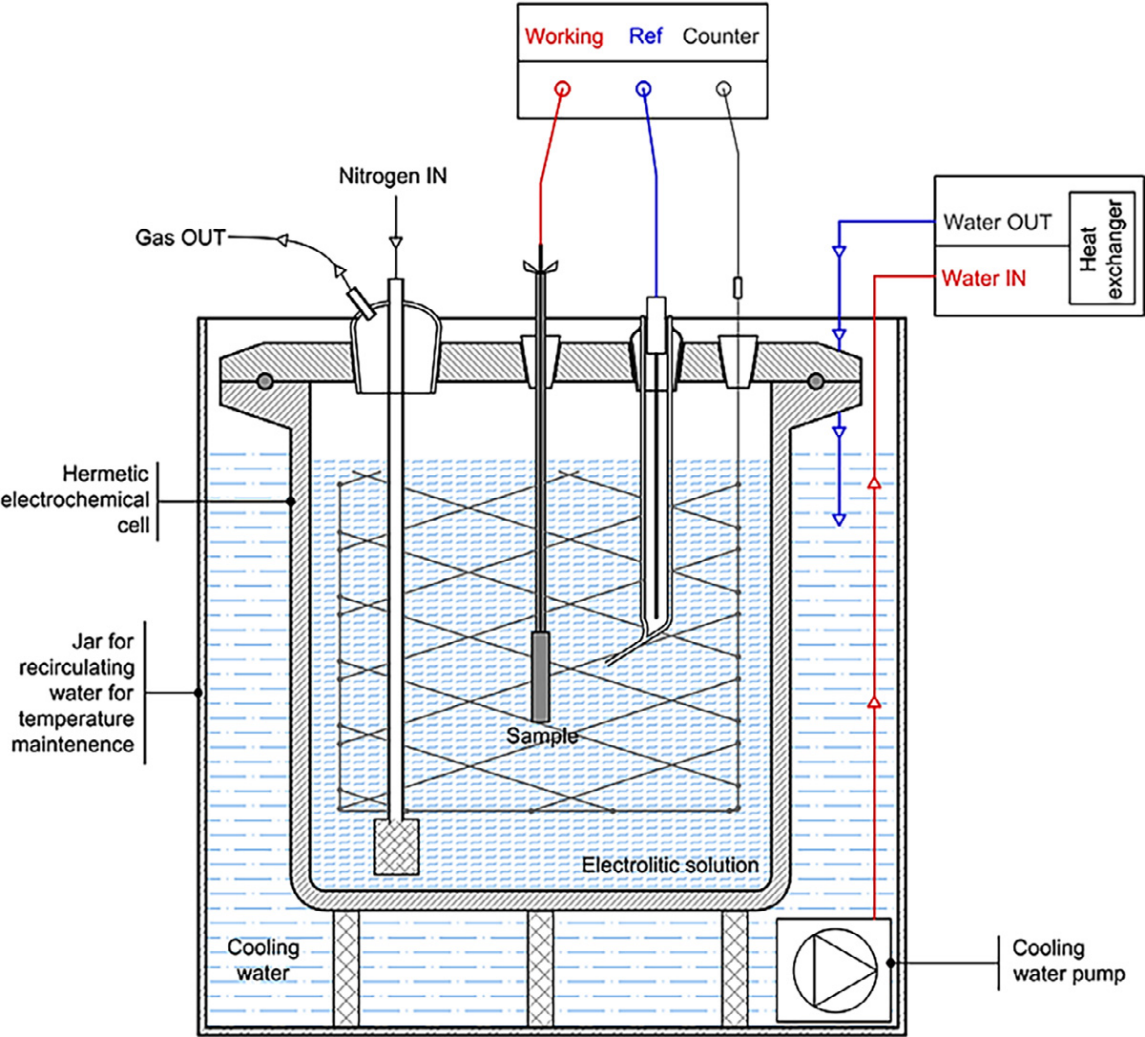
Scheme of the electrochemical charging/discharging cell with a single compartment.

The experimental data was acquired following the procedure described in the work [1]. The tests was performed on cylindrical specimens, with a diameter of 13 mm and a height of 45 mm. The experiments are conduct with the experimental apparatus in Fig. 2. Initially, after sample assembly and nitrogen flushing, the test cell was filled with the carbonate-bicarbonate deaerated solution. The specimens were then polarised at +0.306 V versus SCE to obtain the passivation curve. The passivation potential was assumed to be equal to that recommended by the ISO17081 standard for the detection side of the Devanathan–Stachurski cell operating in the 0.1 M sodium hydroxide solution expressed versus SCE. This potential was also applied to the tests conducted in the carbonate–bicarbonate solution at pH 10. Hydrogen charging was conducted through potentiostatic cathodic polarisation in the range of −1 to −1.8 V versus SCE for 15 h, which was sufficient time for the specimens to achieve a homogeneous hydrogen concentration at a typical value of diffusivity reported for low-carbon steel [3,4] and compared with other techniques like the step potential method and cyclic voltammetry methods [5,6]. During this step, part of the hydrogen produced on the surface was absorbed into the specimen. At the end of the charging phase, polarisation was immediately inverted to apply the anodic polarisation again at +0.306 V versus SCE for 15 h to measure the discharging current curve. The discharging anodic current represents the passivity current enhanced by the current due to the oxidation of hydrogen egressing from the metal.

To generate the pre-processed data, it was necessary following this protocol. It was estimated the net current due to hydrogen oxidation $I_{adj}$, the experimental value of the discharging current was depurated by subtracting the passivation current curve measured during the early phase (1)

(1) $$I_{adj}=I-I_{pass}\;\left[A\right]$$

where I is current measured during discharging step, while $I_{pass}$ is current measured during passivation. The integral sum of this curve, over testing time, allows to estimate charge exchanged $Q_{adj}$. It was possible to evaluate the charge evolution over time $Q_{adj}\left(t_{i}\right)$ as follow in (2)

$$Q_{adj}=\int_{0}^{end}I_{adj}\;dt\;\left[C\right]$$

(2) $$Q_{adj}\left(t_{i}\right)=\sum_{t=0}^{end}Q_{adj}\left(t_{i-1}\right)+\frac{\left(I_{adj}\left(t_{i}\right)+I_{adj}\left(t_{i+1}\right)\right)\cdot \left(t_{i}-t_{i+1}\right)}{2}$$

Therefore, the diffusible hydrogen concentration egress from the sample can be calculate as follow

(3) $$C_{egress,\;adj}\left(t_{i}\right)=\frac{Q_{adj}\left(t_{i}\right)\cdot AW_{H}}{F\cdot m_{samp}}\cdot 10^{6}\;\left[ppm\right]$$

with $AW_{H}$ is the atomic weight of hydrogen, F is Faraday’s constant and $m_{samp}$ is the mass of sample in grams. To estimate the average residual diffusible hydrogen concentration that remain into the sample $C_{res,\;adj}\left(t_{i}\right)$, considering a certain initial concentration at the end of charging, the following evaluation is necessary

(4) $$C_{res,\;adj}\left(t_{i}\right)=C_{egress,\;adj}\left(t=end\right)-C_{egress,\;adj}\left(t_{i}\right)\;\left[ppm\right]$$

where $C_{egress,\;adj}\left(t=end\right)$ is the maximum final value of egress concentration and $C_{egress,\;adj}\left(t_{i}\right)$ is the egress concentration over time. The relative time t and the average residual diffusible hydrogen concentration $C_{egress,\;adj}$ are pre-processed data must be uploaded in the data-driven optimisation MATLABE code.

■ **TARGET CURVE**

The tri-dimensional diffusion of hydrogen from the sample during electrochemical discharge was simulated through the FEM model using *COMSOL Multiphysics* in cylindrical coordinates. The specific cylinder geometry of the sample adopted in this work was considered. The diffusion process in the metal is described by Fick’s second law (5), where C e t are the hydrogen concentration and time, respectively.

(5) $$\left\{ \begin{array}{c} \frac{\partial C}{\partial t}-D\nabla^{2}C=0\\ \begin{array}{c} C_{0}^{*}=\text{constant}\\ D=\text{constant} \end{array}]I.C\\ C_{S}=0\;\text{on}\;\text{surfaces}]B.C. \end{array}\right.$$

The model includes the initial conditions (I.C.) of homogeneous hydrogen concentration $C_{0}^{*}$ and constant diffusion coefficient D. During discharging, the boundary condition (B.C.) on the metal–solution surface imposes the concentration $C_{s}$ to be equal to zero. The simulations conducted varying diffusion coefficients D and initial concentrations $C_{0}^{*}$.

The flux of hydrogen leaving all sample surfaces is then obtained using Fick’s first law (6), given the concentration gradient normal to the external sample surface.

(6) $$\phi_{egress}^{*}=\;-D\cdot \nabla C$$

The mole of egress hydrogen ($n_{egress}^{*}\left(t\right)$) was obtained through the integration of the flux over time. Thus, to obtain the average residual concentration ($C_{res}^{*}$) that remains in the sample at a certain time, considering an initial concentration $C_{0}^{*}$ at the end of charging, the following evaluation is necessary (7):

(7) $$C_{res}^{*}\left(t\right)=\;\sum_{t=0}^{\infty }\;\frac{n_{egress}^{*}\left(t=\infty \right)-n_{egress}^{*}\left(t\right)}{Volume}\;\left[\frac{mol}{m^{3}}\right]$$

About the simulation to discretise the geometry, the axisymmetric property of the problem was exploited. As a result, a mesh was generated over a single rectangular section, with one side aligned along the cylinder's axis. The mesh was automatically generated using a quadrilateral mesh generator, which primarily creates quadrilateral elements. However, in regions of the geometry where it deems necessary, the generator may also introduce triangular elements. In this specific case, the computational plane consisted of 1647 quadrilateral elements. The simulation solved a total of 2009 degrees of freedom, with an integration time step of 60,000 s. It is important to note that the flux was applied over all surfaces. The simulation parameters can be modified depending on the intended application. The values presented here were used to analyse this specific geometry.

To obtain the target curve shown in the data, the results of simulated tests, time $t^{*}$ and residual diffusion hydrogen concentration simulated $C_{res}^{*}\left(t\right)$ must be normalised as describe in [1] introducing the normalised time $\hat{T}$ and normalised concentration ${\hat{C}}^{*}\left(t\right)$ using Eqs. (8) and (9).

(8) $$\hat{T}=\frac{t\cdot D}{r^{2}}$$

(9) $${\hat{C}}^{*}\left(t\right)=\frac{C_{res}^{*}\left(t\right)}{C_{res}^{*}\left(t=0\right)}\equiv \frac{C_{res}^{*}\left(t\right)}{C_{0}^{*}}$$

The diffusion coefficient D and initial hydrogen concentration $C_{0}^{*}$ are that imposed during the simulation, $\hat{T}$ and ${\hat{C}}^{*}\left(t\right)$ formed the target curve (that it is independent by D and $C_{0}^{*}$ as described in [1]).

■ **DATA-DRIVEN OPTIMISATION MATLAB CODE**

The code to elaborate the pre-processed data and target curve to obtain the better D, $C_{0}$ and $t_{0}$ by experimental data, is as follows:

   

## Limitations

In the realisation of the experimental apparatus, it is necessary to ensure a homogeneous distribution of the current field on the specimen during the electrochemical charging and discharging phases in order to guarantee the axial-symmetry and homogeneity conditions imposed in the model assumptions.

There are no particular limitations with regard to the theoretical data of the simulations. In order to obtain a good target curve, it is necessary to create adequate discretisation of the geometry to be simulated so as to have low residuals at the end of the simulation

## Ethics Statement

The authors confirm that they have read and follow the ethical requirements for publication in *Data in Brief*. The current work does not involve human subjects, animal experiments, or any data collected from social media platforms. No ethical approval or informed consent was required, as no experiments or direct data collection from individuals were conducted. All data provided in this article are publicly available or were derived from publicly accessible sources, ensuring full compliance with ethical standards for data sharing. The authors also confirm that all data presented in this manuscript are accurate, properly cited, and do not infringe upon any intellectual property rights.

## CRediT Author Statement

**Luca Gritti:** Methodology, Software, Formal analysis, Investigation, Writing - Original Draft, Visualization; **Denny Coffetti:** Writing - Review & Editing; **Marina Cabrini:** Supervision, Project administration, Conceptualization; **Tommaso Pastore:** Project administration, Conceptualization

## Data Availability

Mendeley DataData in Brief: Experimental Data Supporting Solubility Model (Original data). Mendeley DataData in Brief: Experimental Data Supporting Solubility Model (Original data).
